# Isoliquiritigenin diminishes invasiveness of human nasopharyngeal carcinoma cells associating with inhibition of MMP‐2 expression and STAT3 signalling

**DOI:** 10.1111/jcmm.18586

**Published:** 2024-08-09

**Authors:** Yen‐Ting Lu, Chung‐Han Hsin, Shao‐Hsuan Kao, Yu‐Ting Ho, Fang‐Ling Yeh, Shun‐Fa Yang, Chiao‐Wen Lin

**Affiliations:** ^1^ School of Medicine Chung Shan Medical University Taichung Taiwan; ^2^ Department of Otolaryngology Chung Shan Medical University Hospital Taichung Taiwan; ^3^ Department of Otolaryngology St. Martin De Porres Hospital Chiayi Taiwan; ^4^ Institute of Medicine Chung Shan Medical University Taichung Taiwan; ^5^ Department of Medical Research Chung Shan Medical University Hospital Taichung Taiwan; ^6^ Department of Biochemistry and Molecular Biology University of Massachusetts Amherst Massachusetts USA; ^7^ Institute of Oral Sciences Chung Shan Medical University Taichung Taiwan; ^8^ Department of Dentistry Chung Shan Medical University Hospital Taichung Taiwan

**Keywords:** cancer metastasis, isoliquiritigenin, matrix metalloproteinase‐2, nasopharyngeal carcinoma

## Abstract

Nasopharyngeal carcinoma (NPC) is prevalent in Asia and exhibits highly metastatic characteristics, leading to uncontrolled disease progression. Isoliquiritigenin (ISL) have attracted attention due to their diverse biological and pharmacological properties, including anticancer activities. However, the impact of ISL on the invasive and migratory ability of NPC remains poorly understood. Hence, this study aimed to investigate the in vitro anti‐metastatic effects of ISL on NPC cells and elucidate the underlying signalling pathways. Human NPC cell NPC‐39 and NPC‐BM were utilized as cell models. Migratory and invasive capabilities were evaluated through wound healing and invasion assays, respectively. Gelatin zymography was employed to demonstrate matrix metalloproteinase‐2 (MMP‐2) activity, while western blotting was conducted to analyse protein expression levels and explore signalling cascades. Overexpression of signal transducer and activator of transcription 3 (STAT3) was carried out by transduction of STAT3‐expressing vector. Our findings revealed that ISL effectively suppressed the migration and invasion of NPC cells. Gelatin zymography and Western blotting assays demonstrated that ISL treatment led to a reduction in MMP‐2 enzyme activity and protein expression. Investigation of signalling cascades revealed that ISL treatment resulted in the inhibition of STAT3 phosphorylation. Moreover, overexpression of STAT3 restored the migratory ability of NPC cells in the presence of ISL. Collectively, these findings indicate that ISL inhibits the migration and invasion of NPC cells associating with MMP‐2 downregulation through suppressing STAT3 activation. This suggests that ISL has an anti‐metastatic effect on NPC cells and has potential therapeutic benefit for NPC treatment.

## 1 INTRODUCTION

(NPC) is a malignant tumour arising from the nasopharyngeal mucosal epithelium. Although NPC is a relatively rare cancer globally, it has a remarkably high incidence in certain regions of Southeast Asia and among specific ethnic groups. In 2020, over 130,000 new NPC cases were recorded by the International Agency for Research on Cancer,^1^ and more than 70% of the cases were found in Southeast Asia.^2^ Environmental variables, genetic factors, and Epstein–Barr virus infection play crucial roles in the development of NPC,^2^, ^3^ and the Epstein–Barr virus is linked with 95% of cases of non‐keratinized NPC.^4^ Moreover, NPC cells are highly likely to locally invade lymph nodes and spread to distant sites, causing metastases.^5^, ^6^ After initial treatment, 10%–20% of NPC patients experience local and/or nodular recurrence, leading to poor prognoses. It is imperative to develop new treatments and identify therapeutic targets for NPC.

Detecting NPC early is challenging due to the hidden location of the nasopharynx. Even in areas with high NPC incidence, screening high‐risk populations is reported to improve early detection rates.^7^ Consequently, most NPCs in endemic regions are diagnosed at an advanced stage, with approximately 10% of NPC patients already exhibiting distant metastasis at diagnosis.^2^ The metastatic cascade in NPC often involves cancer cell invasion into surrounding tissues, intravasation into lymphatic or blood vessels, and establishment of secondary tumours in distant organs, such as the liver, bone or lung. Although primary NPC responds well to radiotherapy and chemotherapy, 15%–18.5% of new NPC patients without distant metastasis eventually experience treatment failure due to post‐treatment metastasis of the tumour cells.^8^, ^9^ Metastasis is the primary reason for treatment failure in NPC patients. This challenge is common in combating many cancer types, as metastasis is the leading cause of cancer‐related deaths.^10^, ^11^

Licorice is an ancient medicinal herb, the dried root or rhizome of the plant *Glycyrrhiza Radix*. It possesses many pharmacological activities, including antioxidant, anti‐cancer, anti‐inflammatory and anti‐diabetic activities. These therapeutic properties are attributed to the diverse array of bioactive constituents found within the plant, such as chalcones, isoflavonoids, flavanones, prenyl flavanoid glycycoumarin, triterpene glycyrrhetinic acid and the saponin glycyrrhizin.^12^, ^13^ Among these constituents, isoliquiritigenin (ISL) is an important pharmacological component in Glycyrrhiza root.^14^ ISL has exhibited remarkable antiproliferative activity against various cancer cells alongside its antiangiogenic, anti‐inflammatory, antimicrobial, cardioprotective and immunoregulatory effects.^14^, ^15^, ^16^, ^17^ Recently, increasing studies have explored the promising effects of ISL as an anticancer compound, highlighting its ability to inhibit cell growth of cervical cancer cells through induction of apoptosis,^18^ trigger cell cycle arrest and suppress migration and invasion of prostate cancer cells,^19^, ^20^ and damper the growth of breast cancer cell in xenograft mice.^21^ However, the inhibitory effect of ISL on NPC has not been explored completely.

Dysregulation of various signalling pathways such as signal transducer and activator of transcription (STAT) proteins^22^, ^23^ and expression of proteolytic enzymes such as matrix metalloproteinases (MMPs)^24^, ^25^ has been demonstrated in promoting migratory and invasive capability of cancer cells. MMPs play a crucial role in extracellular matrix (ECM) degradation, enabling cancer cells to breach the basement membrane and invade surrounding tissues.^26^ Among the MMPs, MMP‐2 (gelatinase A) has been particularly implicated in the invasive and metastatic potential of various cancers, including NPC.^27^, ^28^, ^29^, ^30^

Despite the therapeutic potential of ISL, its impact on the migratory and invasive capability of NPC cells remains incompletely understood. Therefore, this study was aimed to investigate the in vitro anti‐metastatic properties of ISL on NPC cells and elucidate the underlying molecular mechanisms. By elucidating the molecular mechanisms underlying ISL's anti‐metastatic effects, our findings could further contribute to the understanding of its pharmacological actions and support its further investigation as a potential adjuvant for NPC treatment.

## 2 MATERIALS AND METHODS

### 2.1 Chemicals and antibodies

ISL (#I3766) and most chemicals were obtained from Sigma‐Aldrich (St. Louis, MO, USA). Primary antibodies which specific recognize human β‐actin (#4967), phospho(p)‐extracellular signal‐regulated kinase (p‐ERK, #9101), total (t)‐ERK (#9102), p‐c‐Jun N‐terminal kinae (p‐JNK, #9251), t‐JNK (#9252), p‐p38 mitogen‐activated protein kinase (p‐p38, #9211), t‐p38 (#9212), focal adhesion kinase (FAK, #3285), p‐FAK(Y397)(#3283), p‐FAK(Y925)(#3284), p‐Src(Y416)(#2101), t‐Src(#2108), p‐STAT3(Y705)(#9131), t‐STAT3(#9139) and matrix metalloproteinase (MMP)‐2(#3852) were acquired from Cell Signaling Technology (Danvers, MA, USA).

### 2.2 Cell culture

The human NPC cell lines NPC‐39, and NPC‐BM were acquired from Dr. M.K. Chen, Changhua Christian Hospital, Changhua, Taiwan. Both cells were cultured in RPMI‐1640 medium (Gibco, Waltham, MA, USA) supplemented with 10% foetal bovine serum (FBS, Gibco, Waltham, MA, USA) and 1% penicillin/streptomycin (Gibco, Waltham, MA, USA). Cells were maintained in a humidified incubator at 37°C with 5% CO_2_. Cells were grown to 80% confluency, and then collected for subsequent treatments.

### 2.3 Cell viability assay

Cell viability were assessed using the 3‐(4,5‐dimethylthiazol‐2‐yl)‐2,5‐diphenyltetrazolium bromide (MTT, Sigma‐Aldrich, St. Louis, MO, USA) assay. Briefly, cells were seeded in 24‐well plates at a density of 5 × 10^4^ cells/mL and allowed to attach overnight. Then, cells were treated with serial concentrations of ISL (0–40 μM) for 24 h. After treatment, the treated cells were washed with phosphate‐buffered saline (PBS) and then incubated with 5 mg/mL MTT solution (Sigma‐Aldrich, St. Louis, MO, USA) for 4 h. The resulting formazan crystals were dissolved in DMSO, and the absorbance at 570 nm was measured using a microplate reader (SpectraMax, Molecular Device, San Jose, CA, USA).

### 2.4 Transwell migration assay and invasion assay

The evaluation of cell migration and invasion was conducted using transwell inserts as previously described.^26^ Briefly, after subjecting the cells to treatment with varying concentrations of ISL (0, 5, 10, 20 and 40 μM) for a duration of 24 h, the surviving NPC cells were collected and seeded onto the upper chamber in a serum‐free medium, while the lower chamber contained medium with 10% FBS as a chemoattractant. For invasion assay, the transwell inserts were pre‐coated with Matrigel (#356235, Corning). Subsequently, the cells were incubated at 37°C for 24 h. Upon completion of the incubation period, the cells were fixed using 100% methanol and stained with a 10% Giemsa solution (#32884, Sigma‐Aldrich, St. Louis, MO, USA) for 3 h. The quantification of cells was performed by employing an Olympus CKX41 microscope (Olympus Corporation, Tokyo, Japan).

### 2.5 Analysis of MMP‐2 activity by gelatin zymography

Gelatin zymography was performed as previously described.^31^ Briefly, conditioned media from ISL‐treated or 0.1% DMSO‐treated cells (control) were collected and concentrated by using Amicon Ultra centrifugal filters (Millipore). Samples were mixed with non‐reducing sample buffer and electrophoresed on 8% SDS‐polyacrylamide gels containing 0.1% gelatin. After electrophoresis, gels were washed and incubated in developing buffer (50 mM Tris–HCl, pH 8.5) at 37°C for 24 h. Gels were then stained with Coomassie Brilliant blue, and areas of gelatinolytic activity appeared as clear bands against a blue background.

### 2.6 Western blotting

Cells were lysed in RIPA buffer containing protease and phosphatase inhibitors. Protein concentrations were determined using the Bradford protein assay (ab119216, abcam, UK). Fifteen microgram crude proteins were separated by SDS‐PAGE, then transferred onto PVDF membranes. After blocking with 5% bovine serum albumin, the membranes were incubated with primary antibodies against human MMP‐2, phosphor(p)‐FAK(Y397), FAK, p‐Src, total(t)‐Src, p‐STAT3(Y705), t‐STAT3, p‐ERK1/2, t‐ERK1/2, p‐p38 MAPK, t‐p38 MAPK and β‐actin overnight at 4°C. After washed with PBS, HRP‐conjugated secondary antibodies were then added, and signals were developed with chemiluminescence and detected and quantitated using a chemiluminescence detection image system (Fuji, Tokyo, Japan).

### 2.7 Transfection of STAT3

NPC‐39 cells at 80% confluence were transfected with a STAT3‐expressing plasmid (STAT3, NM_139276, #SC124125, OriGene, Rockville, MD, USA) with Lipofectamine 3000 (#L3000‐015, Invitrogen, Carlsbad, CA, USA). After 6 h incubation, the STAT3‐expressing plasmid transfected cells were harvested and subjected to transmigration assay.

### 2.8 Statistical analysis

Quantitative data from three independent experiments were presented as mean ± standard deviation (SD). Statistical analyses were conducted using GraphPad Prism software. Differences between groups were analysed using Student's *t*‐test. *p* values <0.05 were considered statistically significant.

## 3 RESULTS

### 3.1 Effect of ISL on NPC cell viability

**FIGURE 1:**
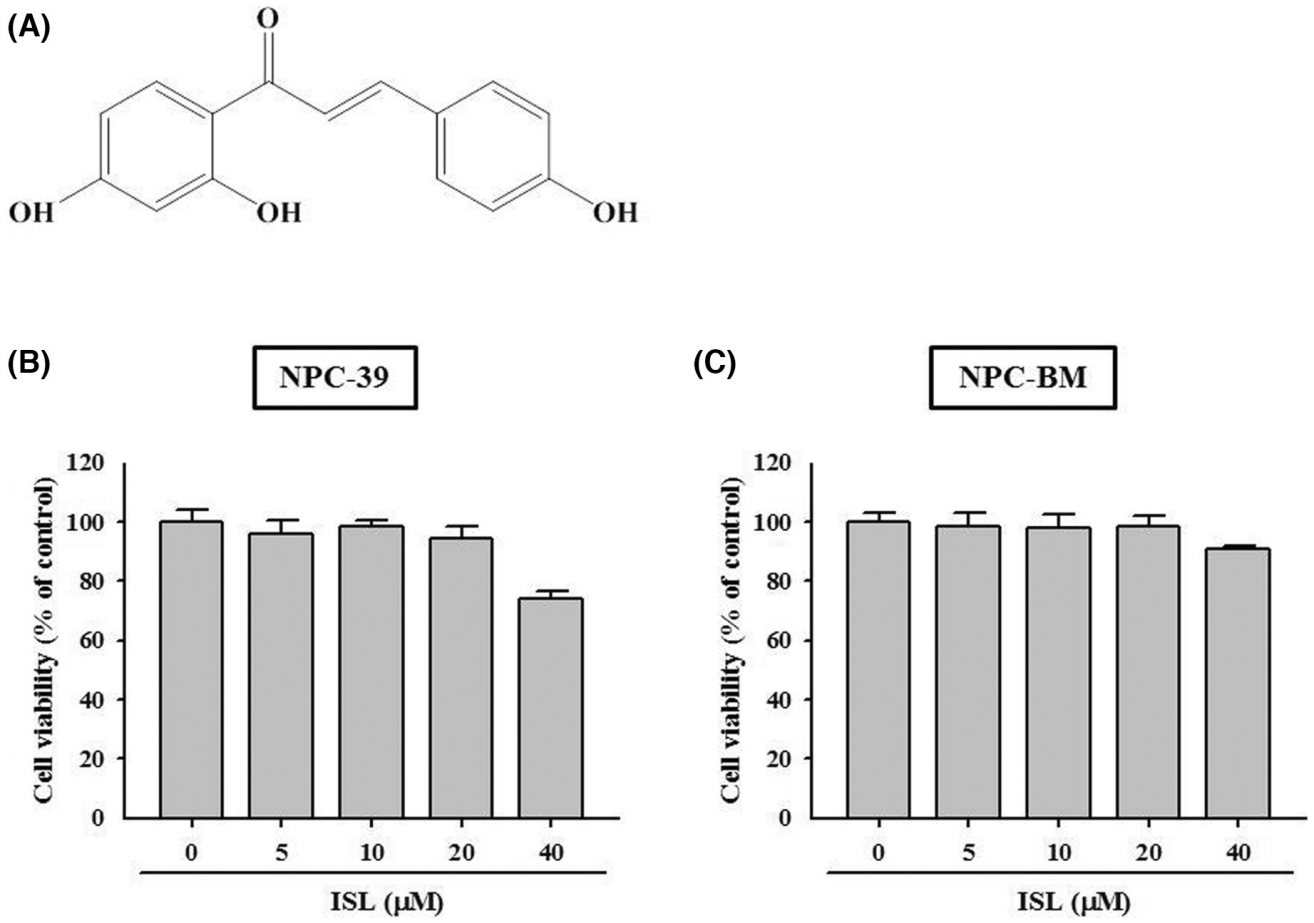
Chemical structure and effect of isoliquiritigenin (ISL) on NPC cells. (A) Chemical structure. (B) NPC‐39 and (C) NPC‐BM cells were exposed to ISL at serial concentrations as indicated for 24 h, and then subjected to viability assay. DMSO (0 μM) was used as sham treatment.

The chemical structure of ISL is shown in Figure 1A, a trihydroxychalcone. The effect of ISL on cell viability of NPC cells was firstly explored. As shown in Figure 1B,C, NPC‐39 and NPC‐BM cells were treated with ISL at 5, 10, 20 and 40 μM for 24 h. Although 40 μM ISL appeared to slightly reduce the cell viability of NPC‐39 cells, the results revealed that the 24 h‐treatments of ISL did not significantly affect the cell viability of NPC‐39 and NPC‐BM cells.

### 3.2 ISL suppressed migratory and invasive ability of NPC cells

**FIGURE 2:**
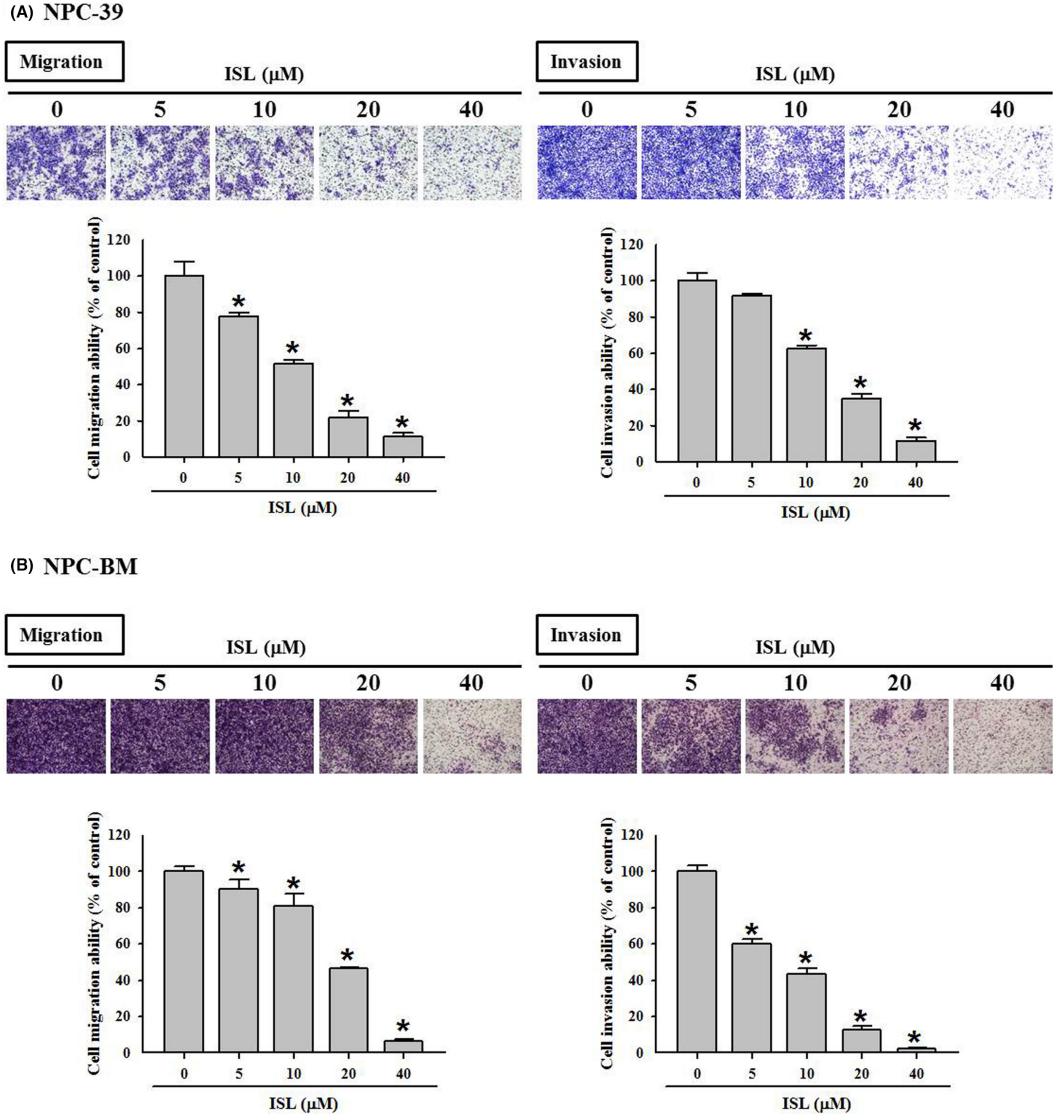
Isoliquiritigenin (ISL) attenuated the migratory and invasive ability of NPC cells. (A) NPC‐39 and (B) NPC‐BM cells were incubated with serial concentrations of ISL as indicated for 24 h, and then subjected to transwell migration assay and invasion assay. The quantitative data were presented as mean ± SD from triplicates independent experiments. *p < 0.05 as compared with sham control (0 μM).

Next, the effects of ISL on migratory and invasive ability of NPC cells were investigated. Our results showed that ISL dose‐dependently attenuated the cell migration and invasion of NPC‐39 cells by employing transmigration assay and invasion assay (Figure 2A). With 24 h ISL treatment at 40 μM, the average numbers of migrated and invaded cells were reduced to 11.3% and 11.8% of control, respectively (*p* < 0.05). Similarly, ISL exerted significant inhibitory effects on migratory and invasive ability on NPC‐BM cells (Figure 2B), and 40 μM ISL treatments reduced the average numbers of migrated and invaded cells to 6.3% and 2.5% of control, respectively (*p* < 0.05). Taken together, these findings indicate that ISL can significantly suppress the migratory and invasive ability of NPC cells.

### 3.3 ISL downregulated MMP‐2 activity and expression in NPC cells

**FIGURE 3:**
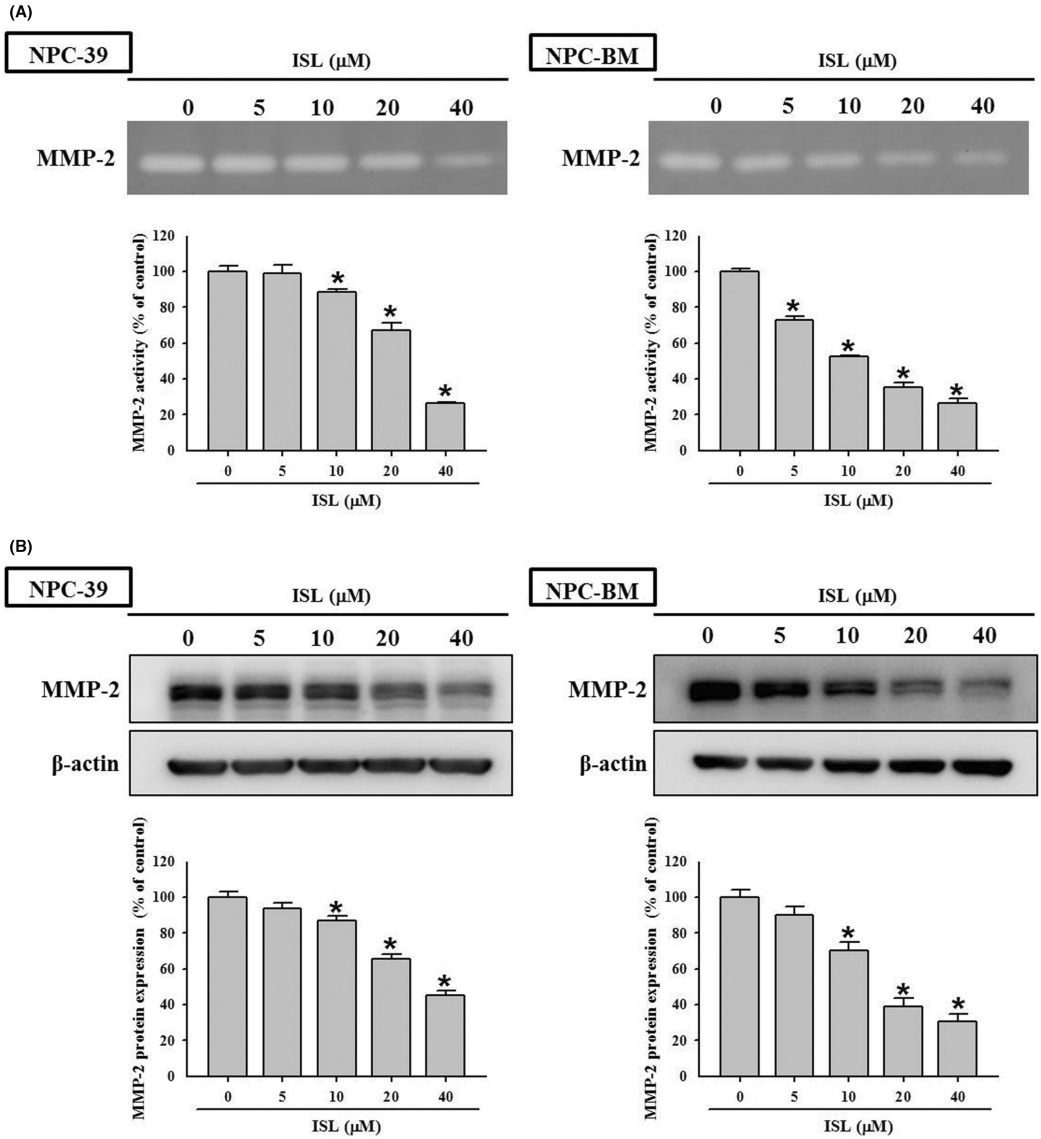
Isoliquiritigenin (ISL) reduced the activity and the expression of MMP‐2 in NPC cells. (A) NPC‐39 and (B) NPC‐BM cells were incubated with serial concentrations of ISL as indicated for 24 h, and then the cultured medium and treated cells were subjected to gelatin zymography analysis for MMP‐2 activity and western blotting for MMP‐2 expression, respectively. The quantitative data were presented as mean ± SD from triplicates independent experiments. *p < 0.05 as compared with sham control (0 μM).

MMP‐2 plays a key role in proliferation and invasiveness of NPC cells.^28^, ^32^ Therefore, whether ISL modulates expression of MMP‐2 is further studied. As shown in Figure 3A, the activity of MMP‐2 produced by NPC‐39 and NPC‐BM cells was assessed by using gelatin zymography assay, and the results exhibited that ISL dose‐dependently diminished the gelatinolytic activity of MMP‐2 in culture medium to 26.6% and 25.9% of control, respectively (*p* < 0.05 compared with control). Then, the expression of MMP‐2 in NPC cells was determined by western blotting, and the results revealed that ISL significantly downregulated the MMP‐2 expression in both NPC‐39 and NPC‐BM cells to 45.4% and 30.4% in a dose‐dependent fashion, respectively (*p* < 0.05 compared with control). Collectively, these findings reveal that ISL downregulates the production and expression of MMP‐2 in NPC cells.

### 3.4 ISL modulates STAT3 signalling pathway in NPC cells

**FIGURE 4:**
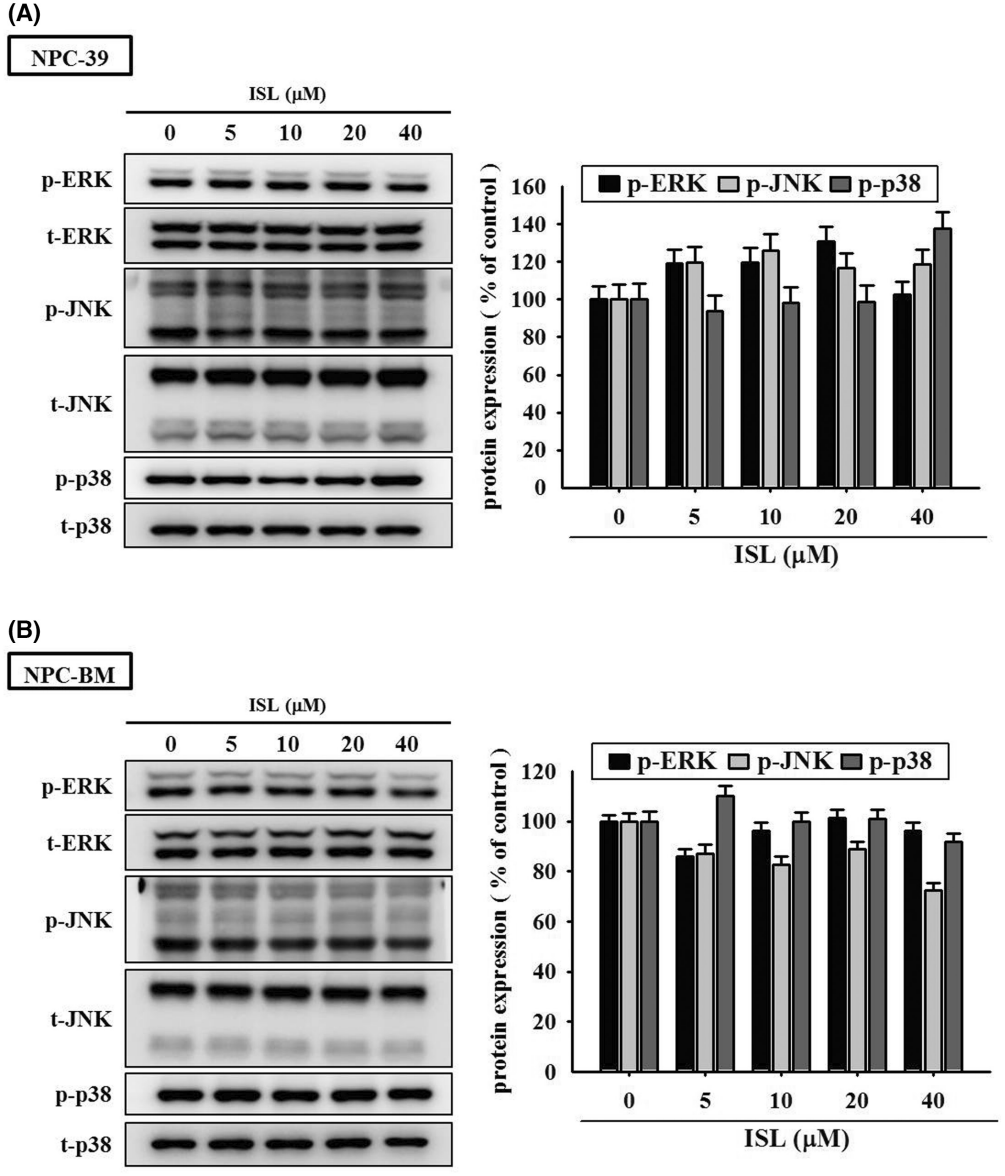
Effects of isoliquiritigenin (ISL) on MAPK signalling cascades in NPC cells. (A) NPC‐39 and (B) NPC‐BM cells were incubated with serial concentrations of ISL as indicated for 4 h, and then the treated cells were lysed and subjected to Western blotting for individual signalling components. Phosphorylated protein and total protein represented active form and internal control, respectively. Relative signal density (ERK1/2, JNK1/2 and p38) was assessed using densitometric analysis. The quantitative data were presented as mean ± SD from triplicates independent experiments.

**FIGURE 5:**
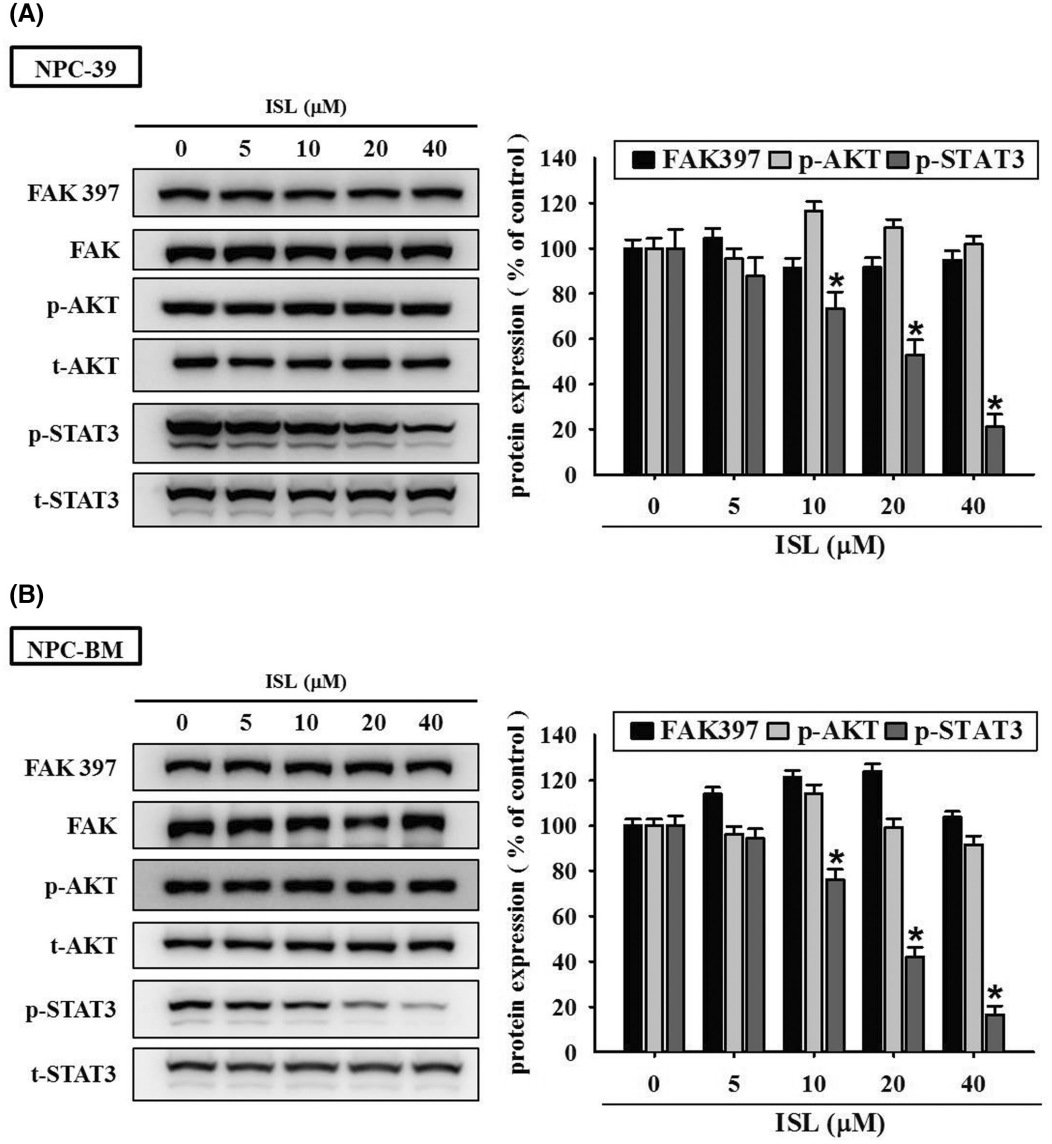
Effects of Isoliquiritigenin (ISL) on FAK, AKT and STAT3 signalling cascades in NPC cells. (A) NPC‐39 and (B) NPC‐BM cells were incubated with serial concentrations of ISL as indicated for 4 h, and then the treated cells were lysed and subjected to western blotting for individual signalling components. Phosphorylated protein and total protein represented active form and internal control, respectively. Relative signal density (FAK, AKT and STAT3) was assessed using densitometric analysis. The quantitative data were presented as mean ± SD from triplicates independent experiments. *p < 0.05 as compared with sham control (0 μM).

Previous studies have demonstrated the association of FAK, STAT3 and MAPK signalling pathways with invasiveness of NPC cells.^6^, ^26^, ^32^, ^33^ In order to elucidate the underlying molecular mechanisms, we examined the effect of ISL on these signalling pathways by employing western blotting analysis. Our findings revealed that no significant changes were observed in the phosphorylation levels of ERK1/2, JNK1/2 and p38 after ISL treatment in the NPC‐39 and NPC‐BM cells (Figure 4A,B). However, ISL treatment led to a dose‐dependent inhibition of STAT3 phosphorylation in NPC‐39 (Figure 5A) and NPC‐BM cells (Figure 5B). On the contrary, no significant changes were observed in the phosphorylation levels of FAK(Y397), and AKT signal pathway. Together, these observations suggest that ISL may specifically target the STAT3 signalling pathway.

### 3.5 Overexpression of STAT3 restored the migratory ability of NPC‐39 cells with exposure to ISL

To verify the role of STAT3 signalling in ISL's inhibition of NPC cell invasiveness, we established a STAT3‐overexpressing NPC cell line by transfection with a STAT3 expression vector. As shown in Figure 5A,B, consistently, 20 μM ISL significantly diminished the MMP‐2 protein expression and cell migratory ability of NPC‐39 and NPC‐BM cell. Notably, the MMP‐2 protein expression and cell migratory ability were significantly promoted in STAT3‐overexpressing NPC cells as compared to its parental NPC cells (*p* < 0.05). In addition, the MMP‐2 protein expression and cell migratory ability diminished by ISL was restored in STAT3‐overexpressing NPC cells (*p* < 0.05 compared with parental NPC cells). Collectively, these findings demonstrate that STAT3 signalling is involved in the inhibitory activity of ISL on NPC cells.

## 4 DISCUSSION

The present study unveils the potent anti‐metastatic effects of the natural compound ISL against NPC cells and sheds light on the underlying molecular mechanisms. Our findings demonstrate that ISL effectively inhibits the migratory and invasive capabilities of NPC cells in a dose‐dependent manner. Mechanistic investigations reveal that the attenuation of NPC cell invasiveness by ISL, associating with the downregulation of MMP‐2 expression and activity, is modulated by the suppression of the STAT3 signalling pathway.

**FIGURE 6:**
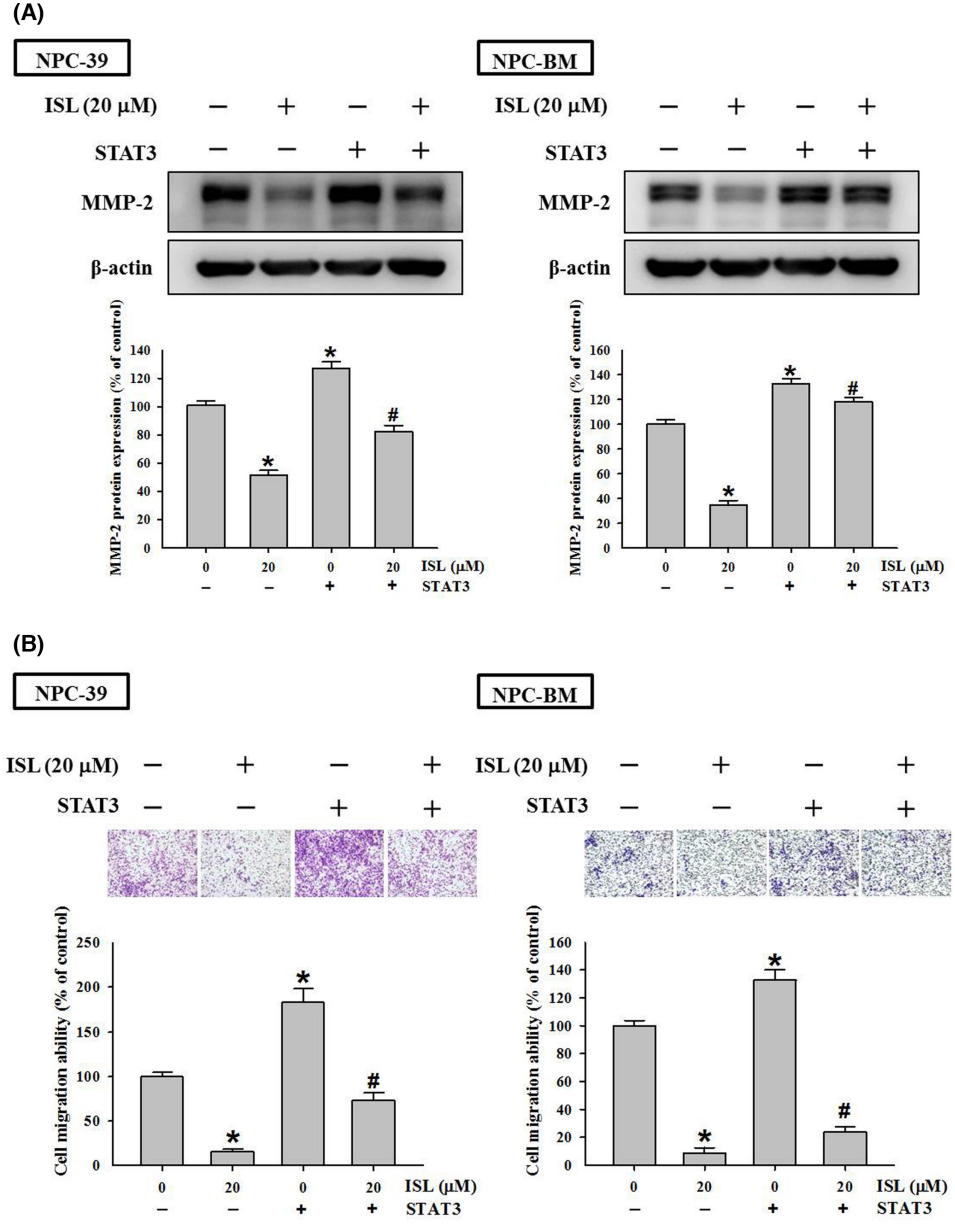
Involvement of STAT3 in isoliquiritigenin (ISL)‐inhibited MMP‐2 protein expression and cell migratory ability of NPC cells. NPC‐39 and NPC‐BM cells were transfected with control vector or STAT3‐expressing vector for 48 h, incubated with 20 μM ISL for 24 h, and then subjected to (A) western blotting assay and (B) cell migration assay. The cell migration ability was expressed as the percentage of migrated cells in the control group. The quantitative data were presented as mean ± SD from triplicates independent experiments. *, *p* < 0.05 as compared with sham control (0 μM). ^#^ *p* < 0.05 as compared with ISL treatment (20 μM).

During the process of metastasis, MMP‐2, a zinc‐dependent endopeptidase, plays a crucial role in ECM degradation organization and cell migration by activating remodelling and path‐clearing processes.^34^ Numerous studies have discovered that the elevated levels of MMP‐2 are the determinants of the aggressive behaviour of tumour cells.^35^ This finding highlights the significance of monitoring MMP‐2 levels in cancer patients to better predict the progression and plan the appropriate treatment. Furthermore, by using integrated proteomics analyses, increased production of MMP‐2 and integrin complexes mediated by activation of focal adhesion kinase (FAK) signalling has been shown to contribute to the high aggressiveness of metastatic colorectal cancer.^36^ Our results show that ISL downregulates MMP‐2 expression and enzymatic activity in NPC cells, suggesting that ISL may possess broad anti‐metastatic potential by targeting MMP‐2 across various malignancies. (Figure 6).

STAT3 is recognized as an oncogene, with dysregulated STAT3 activity reported in nearly 70% of cancers.^37^ The constant activation of STAT3 has been documented across various tumour types, facilitated by mechanisms such as the hyperactivation of receptors for pro‐oncogenic cytokines and growth factors, the loss of negative regulation, and heightened cytokine stimulation.^38^ In NPC, STAT3 plays a pivotal role in tumour progression, metastasis, and therapeutic resistance.^39^ Our observations show that ISL inhibits STAT3 phosphorylation in NPC cells, coupled with the finding that ectopic overexpression of STAT3 restores the migratory ability of ISL‐treated cells, strongly implicates the STAT3 pathway as a crucial target of ISL's anti‐metastatic effects. These results are consistent with previous reports demonstrating the ability of ISL to modulate STAT3 signalling in NPC and other cancer models. Mounting research has demonstrated a strong link between the STAT3 signalling cascade and the regulation of matrix metalloproteinase expression and activation, notably MMP‐2, in cancer cells.^40^, ^41^ Therefore, it is plausible that the ISL‐mediated downregulation of MMP‐2 observed in our study is a downstream consequence of STAT3 inhibition. Moreover, our observations show that no significant changes were observed in the phosphorylation levels of ERK1/2, JNK1/2 and p38 after ISL treatment in the NPC cells. Luo et al., reported that ISL can inhibit the growth of colorectal cancer cells by inhibiting of the PI3K/Akt signalling pathway.^42^ Further investigations are warranted to delineate the precise mechanisms by which ISL modulates STAT3 signalling and the potentialnvolvement of other signalling cascades in mediating its anti‐metastatic effects in NPC cells.

Despite these limitations, our findings highlight the potential of ISL as a therapeutic agent for targeting NPC metastasis. The ability of ISL to suppress NPC cell migration and invasion, at least partially through the inhibition of the STAT3/MMP‐2 axis, provides a rationale for further evaluation of this compound in preclinical model of metastatic NPC. Given the significant morbidity and mortality associated with metastatic NPC, the development of novel therapeutic strategies targeting metastasis is of paramount importance, and our study contributes to this endeavour by identifying ISL as a potential candidate.

## AUTHOR CONTRIBUTIONS

**Yen‐Ting Lu:** Conceptualization (equal); writing – original draft (equal); writing – review and editing (equal). **Chung‐Han Hsin:** Conceptualization (equal); writing – original draft (equal). **Shao‐Hsuan Kao:** Methodology (equal); writing – original draft (equal). **Yu‐Ting Ho:** Methodology (equal). **Fang‐Ling Yeh:** Methodology (equal). **Shun‐Fa Yang:** Conceptualization (equal); writing – original draft (equal); writing – review and editing (equal). **Chiao‐Wen Lin:** Conceptualization (equal); writing – original draft (equal); writing – review and editing (equal).

## CONFLICT OF INTEREST STATEMENT

The authors declare that no competing interests exist.

## Data Availability

The data used to support the findings of the present study are available from the corresponding author upon request.
